# Adaptively evolved *Escherichia coli* for improved ability of formate utilization as a carbon source in sugar-free conditions

**DOI:** 10.1186/s13068-019-1547-z

**Published:** 2019-09-03

**Authors:** Seung-Jin Kim, Jihee Yoon, Dae-Kyun Im, Yong Hwan Kim, Min-Kyu Oh

**Affiliations:** 1 Department of Chemical and Biological Engineering, Korea University, Seongbuk-gu, Seoul, 02841 Republic of Korea; 2 School of Energy and Chemical Engineering, UNIST, Ulju-gun, Ulsan, 44919 Republic of Korea

**Keywords:** Formate, *Escherichia coli*, Adaptive laboratory evolution, Carbon-labeling experiment

## Abstract

**Background:**

Formate converted from CO_2_ reduction has great potential as a sustainable feedstock for biological production of biofuels and biochemicals. Nevertheless, utilization of formate for growth and chemical production by microbial species is limited due to its toxicity or the lack of a metabolic pathway. Here, we constructed a formate assimilation pathway in *Escherichia coli* and applied adaptive laboratory evolution to improve formate utilization as a carbon source in sugar-free conditions.

**Results:**

The genes related to the tetrahydrofolate and serine cycles from *Methylobacterium extorquens* AM1 were overexpressed for formate assimilation, which was proved by the ^13^C-labeling experiments. The amino acids detected by GC/MS showed significant carbon labeling due to biomass production from formate. Then, 150 serial subcultures were performed to screen for evolved strains with improved ability to utilize formate. The genomes of evolved mutants were sequenced and the mutations were associated with formate dehydrogenation, folate metabolism, and biofilm formation. Last, 90 mg/L of ethanol production from formate was achieved using fed-batch cultivation without addition of sugars.

**Conclusion:**

This work demonstrates the effectiveness of the introduction of a formate assimilation pathway, combined with adaptive laboratory evolution, to achieve the utilization of formate as a carbon source. This study suggests that the constructed *E. coli* could serve as a strain to exploit formate and captured CO_2_.

## Background

The increased level of atmospheric carbon dioxide (CO_2_) is the main cause of global warming. Accordingly, carbon dioxide capture and storage (CCS) technology is considered as an important research area for a sustainable environment. Among the options available, hydrogen-dependent conversion of CO_2_ into formate has the advantage of storing and transporting hydrogen as well as utilizing captured CO_2_ [1–5]. These reactions of CO_2_ reduction have been extensively studied using both chemical and biological catalysts, facilitating an easier approach for formate production [6–9]. In particular, cost-effective formate production can be regarded as a potential way of sequestering CO_2_ [10–12]. This in turn has drawn attention to formate as a promising carbon source for use in the biological production of useful chemicals [13–16]. Although native formatotrophic microbes are able to convert formate into biomass or biochemicals, utilization of formate as a carbon source in bioprocesses is limited owing to technical difficulties in the genetic modification of native formatotrophs or because of their low biomass and product yields [5]. It is therefore crucial to focus on commonly used industrial organisms that have higher growth rates and are easy to genetically manipulate for formate consumption. For example, metabolic engineering has been recently attempted in *Escherichia coli* to increase formate fixation abilities, because of the ease of genetic manipulation in this species [17, 18].

**Fig. 1:**
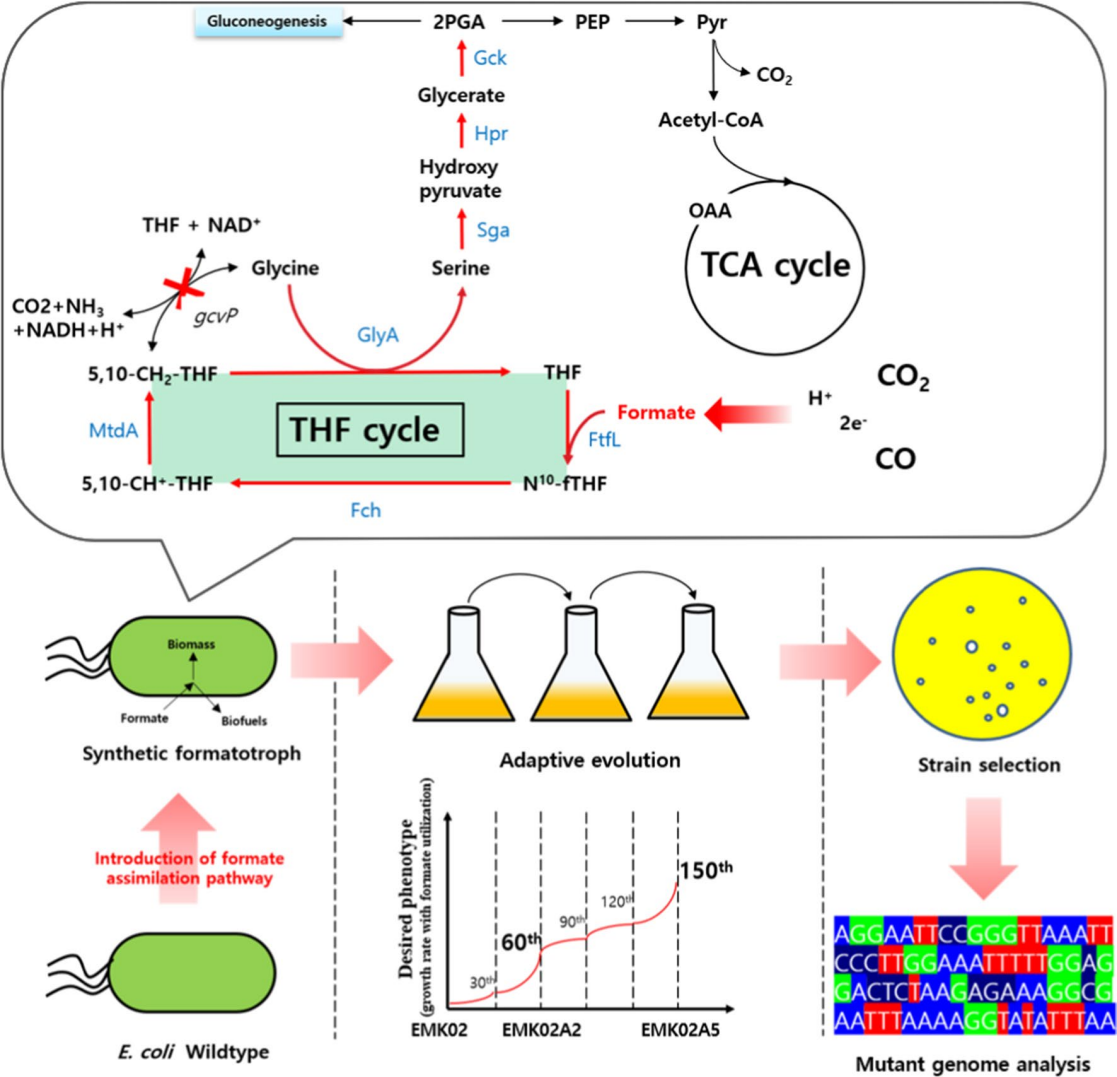
Scheme of *E. coli*-based synthetic formatotrophic strain development. The formate assimilation pathways were constructed in *E. coli* and adaptive laboratory evolution was carried out with 150 serial subcultures. The red arrows indicate engineered pathways and the black arrows indicate innate pathways. THF, tetrahydrofolate; N^10^-fTHF, 10-formyl tetrahydrofolate; 5,10-CH^+^-THF, 5,10-methenyl tetrahydrofolate; 5,10-CH_2_-THF, 5,10-methylene tetrahydrofolate; 2PGA, 2-phosphoglycerate; PEP, phosphoenolpyruvate; Pyr, pyruvate; OAA, oxaloacetate; FtfL, formate-tetrahydrofolate ligase; Fch, methenyl tetrahydrofolate cyclohydrolase; MtdA, methylene-tetrahydrofolate dehydrogenase; GlyA, serine hydroxymethyltransferase; Sga, serine-glyoxylate transaminase; Hpr, hydroxypyruvate reductase; Gck, glycerate kinase

In this study, we developed *E. coli* mutant strains capable of utilizing formate as a carbon source in sugar-free conditions, through the introduction of the tetrahydrofolate cycle and serine utilizing pathway genes (Fig. 1). This pathway was chosen because the enzymes in the pathway are oxygen-tolerant and serine can be easily accessible to the central carbon metabolism [14, 19]. Therefore, the related genes were cloned from *Methylobacterium extorquens* AM1 and overexpressed. In addition, adaptive laboratory evolution (ALE) [20, 21] was carried out until the strain could utilize formate and grow at a significant rate. After conducting the ALE experiment for 150 serial subcultures, mutant strains with the desired phenotype were screened for and their genomes were sequenced. Based on the genome sequences, a few mechanisms, possibly responsible for increased formate utilization and resistance to formate toxicity, were examined. Finally, the resulting *E. coli* were engineered to convert formate into ethanol.

## Results and discussion

### Construction of a formate assimilation pathway in *E. coli*

**Fig. 2:**
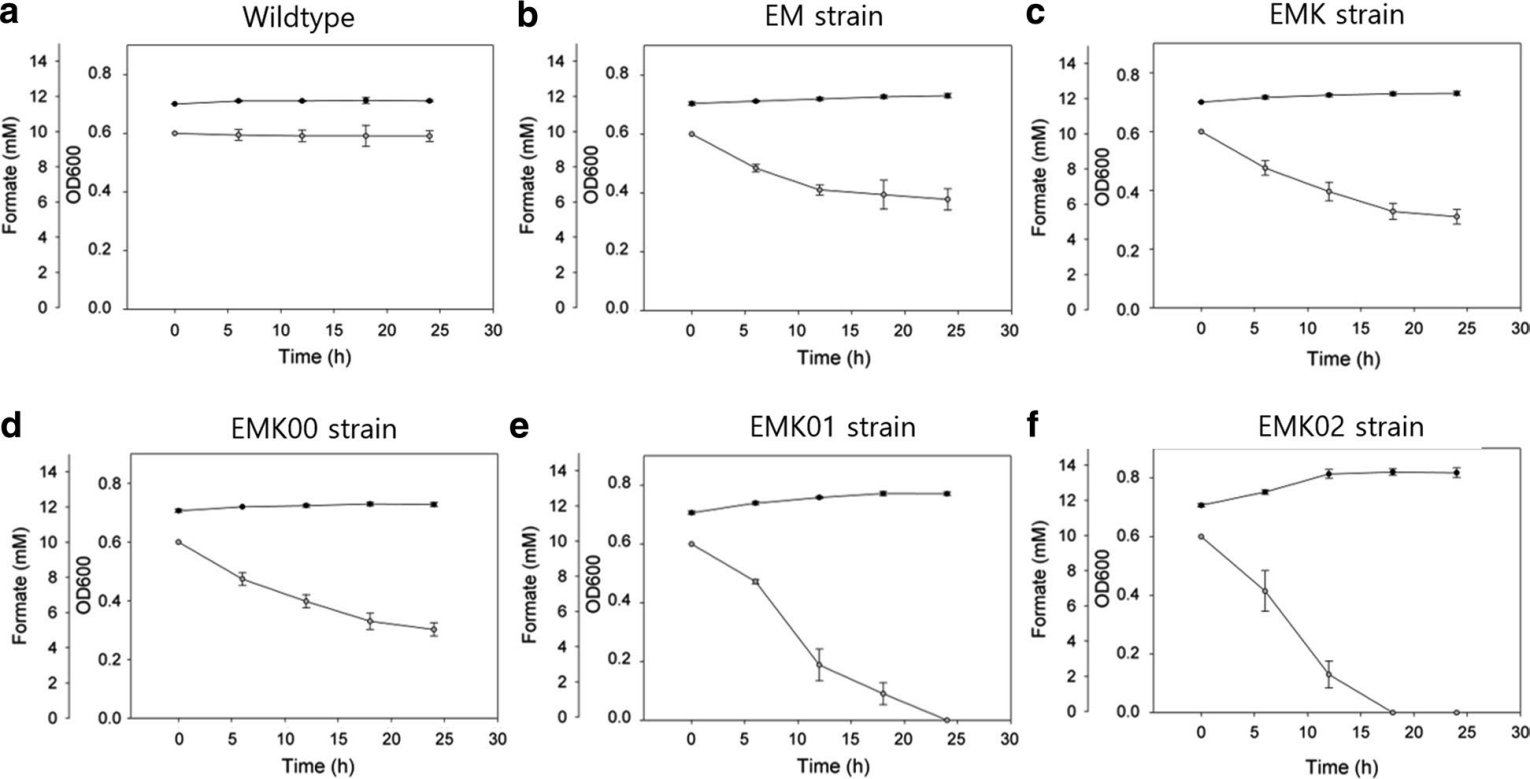
Formate utilization and biomass production at various stages of *E. coli* strain development with iOD of 0.7. The strains grew in formate M9 minimal medium (**a**–**c**) and formate M9 minimal medium supplemented with 1 g/L of glycine (**d**–**f**). Wild-type: *Escherichia coli* BL21; EM: FtfL overexpression in *E. coli*; EMK: FtfL, MtdA and Fch overexpression in *E. coli*; EMK00: *gcvP* knockout in the EMK strain; EMK01: GlyA overexpression in the EMK00 strain; EMK02: Sga, Hpr, and Gck overexpressions in the EMK01 strain; Black circles: Optical density at 600 nm (OD600); White circles: formate concentrations

The serine cycle pathway, one of the formate assimilation pathways, has the advantage that the serine is relatively easily accessible to central carbon metabolism and the enzymes involved in this serine utilizing pathway show oxygen tolerance [19]. Therefore, we attempted the construction of three pathway modules to implement formate assimilation through the serine utilizing pathway in *E. coli* (Fig. 1) (i) the THF (tetrahydrofolate) cycle [15, 24] composed of formate-tetrahydrofolate ligase (FtfL), methenyl tetrahydrofolate cyclohydrolase (Fch), and methylene-tetrahydrofolate dehydrogenase (MtdA); (ii) the serine synthesis enzyme, serine hydroxymethyltransferase (GlyA), from 5,10-CH_2_-THF and glycine [15, 25]; and (iii) the pathway converting serine to phosphoglyceric acid (PGA) for bacterial growth, comprising serine-glyoxylate transaminase (Sga), hydroxypyruvate reductase (Hpr), and glycerate kinase (Gck). After the insertion of these modules, *E. coli* strains were cultured in formate M9 minimal medium to test their ability to utilize formate as a carbon source (Fig. 2). Because no detectable biomass formation was observed when inoculations were made at an OD of 0.1 (data not shown), the initial optical density (iOD) was increased to 0.7.

The wild-type strain did not show any formate assimilation ability, resulting in no biomass formation (Fig. 2a). When FtfL from *M. extorquens* AM1 was overexpressed, some extent of formate utilization was observed (Fig. 2b). The EMK strain, overexpressing the three genes of the THF cycle, *ftfL*, *fch*, and *mtdA* from *M. extorquens* AM1, was able to assimilate formate but only at a very low level (Fig. 2c). The insertion of the second and third modules in this strain did not result in an additional increase in formate assimilation (data not shown), possibly because the methyl unit in the THF cannot be converted to serine efficiently in *E. coli*.

To resolve this limitation, *gcvP* (Gene ID: 947,394 [Genbank]), encoding one of the components of the glycine cleavage system (GCS) [26, 27], was deleted. The resulting strain, EMK00, displayed similar formate utilization compared with its parental strain when glycine was supplied in the medium to induce serine biosynthesis [18] (Fig. 2d). To enhance the efficiency of the serine synthesis, *glyA* was cloned from *M. extorquens* and overexpressed (EMK01 strain), which resulted in an increased ability to utilize formate compared to the EMK00 strain (Fig. 2e). In addition, the third module, based on the *sga, hpr,* and *gck* genes from *M. extorquens*, was introduced into EMK01 to convert serine into PGA (Fig. 2f). Even though this strain (EMK02) showed higher formate uptake and growth rates compared to the other strains, its growth was still very limited.

**Fig. 3:**
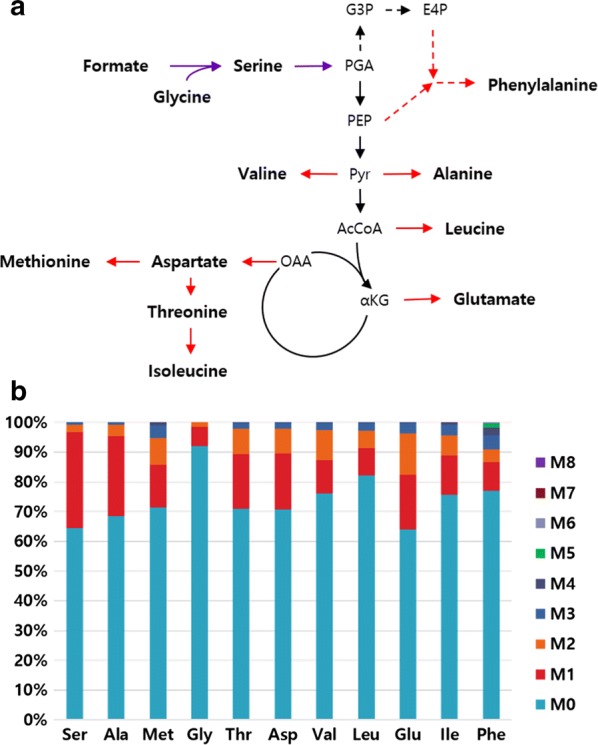
Carbon-labeling experiment with ^13^C-labeled formate in the EMK02 strain. The pathway involved in the synthesis of amino acids in *Escherichia coli* (**a**). OAA, oxaloacetate; AcCoA, acetyl coenzyme A; Pyr, pyruvate; αKG, α-ketoglutarate; PEP, phosphoenolpyruvate; PGA, phosphoglycerate; G3P, glycerate 3-phosphate; E4P, erythrose 4-phosphate; Red arrows: amino acid synthetic pathway; Purple arrows: constructed formate assimilation pathway. The proportion of labeled amino acids in the EMK02 strain after 6-h cultivation with ^13^C-labeled formate in formate M9 minimal medium supplied with 1 g/L glycine at iOD of 0.7 (**b**). Mass isotopomer distribution is displayed in the stacked bar graph and M0–M8 denotes the number of incorporated ^13^C carbon atoms in proteinogenic amino acids

To make sure that formate was used to produce biomass through the introduction of a formate assimilation pathway, we did ^13^C-labeling experiments [28, 29]. Of the 20 amino acids, 11 were detected by GC/MS, which showed significant carbon labeling due to biomass production from formate. The pathways involved in the synthesis of the 11 amino acids in *E. coli* are shown in Fig. 3a. When ^13^C-labeled formate was supplied to the M9 minimal medium with 1 g/L glycine and amino acids from biomass were analyzed by GC/MS in the EMK02 strain, significant proportions of amino acids [methionine (28.4%), threonine (29.1%), serine (35.5%), aspartate (29.1%), glutamate (36.1%), alanine (31.6%), etc.] (Fig. 3b)] contained ^13^C. Since the culture was initiated with an iOD of 0.7, a significant proportion of amino acids were derived from the inoculated cells, resulting in a high M0 proportion. Nevertheless, these ^13^C-labeling experiment results demonstrated that the *E. coli* strain with the serine utilizing pathway inserted was able to convert formate into biomass.

### Adaptive laboratory evolution (ALE) of EMK02

**Fig. 4:**
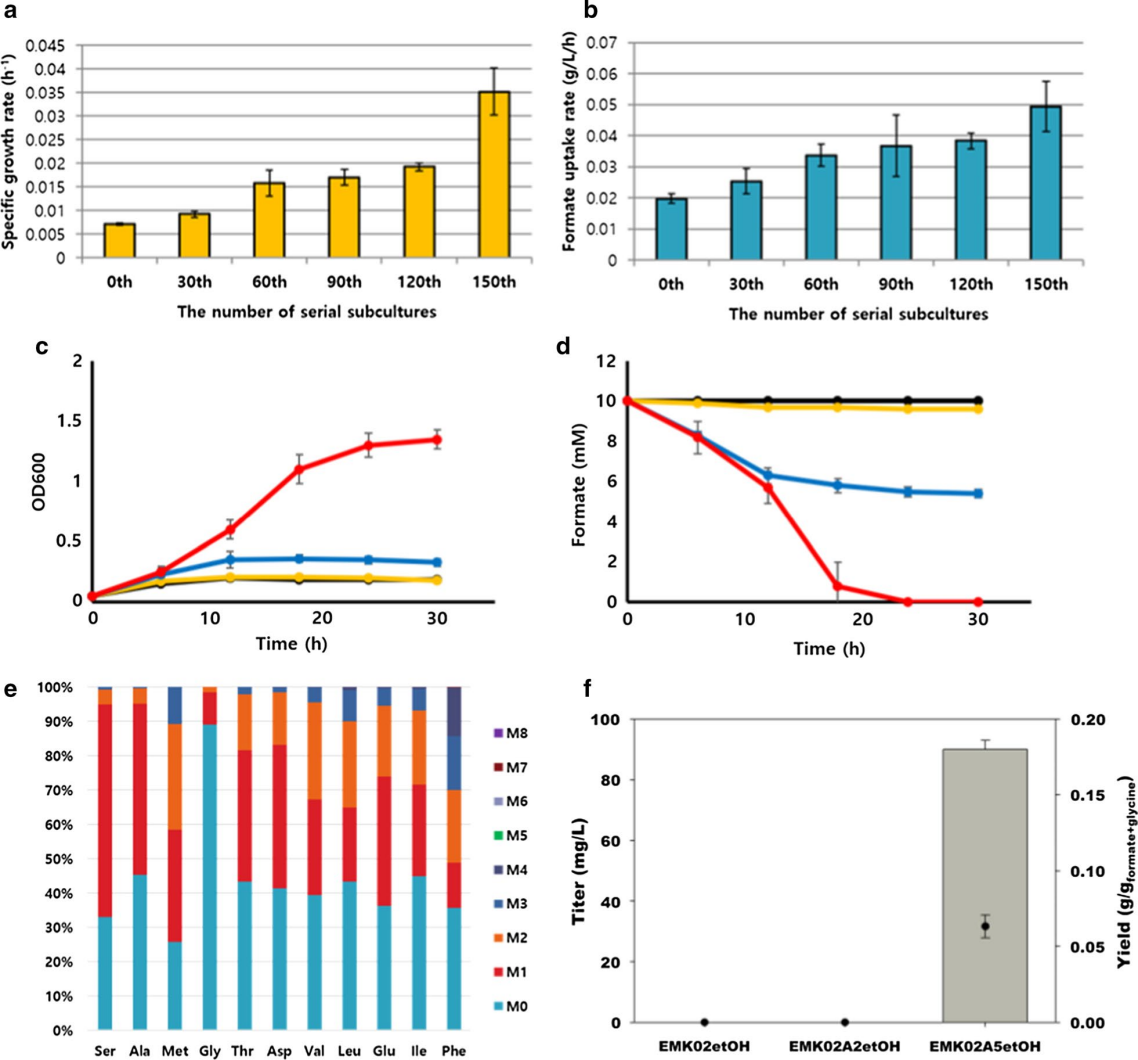
Formate uptake and specific growth rates of ALE mutants. The specific growth rate (**a**) and formate uptake rate (**b**) for screened ALE evolved strains, growing in formate M9 minimal medium supplied with glycine at iOD of 0.7. Comparison of bacterial growth (**c**) and formate utilization (**d**) of wild-type (BL21) (black), EMK02 (yellow), EMK02A2 (blue), and EMK02A5 (red) in EMK medium at iOD of 0.1. The fractions of labeled amino acids in the EMK02A5 strain after 18-h cultivation with ^13^C-labeled formate at iOD of 0.1 (**e**). Ethanol production in different strains (**f**). Bars and dots represent titer and yield, respectively. The strains, EMK02etOH, EMK02A2etOH, and EMK02A5etOH, represented EMK02, EMK02A2, EMK02A5 harboring plasmid for overexpression of pyruvate decarboxylase and alcohol dehydrogenase for ethanol production, respectively. The detailed description of strains is given in Table 1

The EMK02 strain, in which the formate assimilation pathway was established, still showed low formate utilization. To overcome this limitation, adaptive laboratory evolution (ALE) [20, 30] was carried out until the desired phenotypes emerged. The EMK02 strain was cultured in modified EMK medium. Small amounts of yeast extract and glycine were supplied in order to achieve sufficient bacterial growth to subculture once every 24 h. The amount of yeast extract was gradually reduced, while the amount of formate was progressively augmented to enhance the formate utilization ability (Fig. 1). Adaptive laboratory evolution was carried out by 150 serial subcultures and strains with the highest growth rate were selected at every 30th subculture. Formate uptake and growth rates of the strains, as assessed every 30th subculture, gradually increased and the strains selected after the 60th and 150th serial subcultures showed significantly higher formate uptake and growth rates than their ancestors (Fig. 4a, b). These strains were named as EMK02A2 and EMK02A5, respectively (Table 1).

**Table 1:** Bacterial strains and plasmids used in this study

Name	Description	Reference
Strains		
DH5α		RBC
Wild-type	BL21(DE3)	KCTC
EM	BL21(DE3) harboring pZAM	This study
EMK	EM harboring pZAM01	This study
EMK00	EMK Δ*gcvP*	This study
EMK01	EMK00 harboring pZAM02	This study
EMK02	EMK00 harboring pZAM02 and pCDM02	This study
EMK02A2	ALE mutant from EMK02 for 60th serial subcultures	This study
EMK02A5	ALE mutant from EMK02 for 150th serial subcultures	This study
vA5f	EMK02A2 harboring pZSfimCD	This study
vA5y	EMK02A2 harboring pZSydeH	This study
vA5h	EMK02A2 harboring pZShtrE	This study
vA5c	EMK02A2 harboring pZScsgD	This study
EMK02etOH	EMK02 harboring pZSetOH	This study
EMK02A2etOH	EMK02A2 harboring pZSetOH	This study
EMK02A5etOH	EMK02A5 harboring pZSetOH	This study
Plasmids		
pZA31MCS	p15A ori PLtetO-1 CmR	Expressys
pZS21MCS	pCS101 ori PLtetO-1 KanR	Expressys
pCDFDuet-1	Double T7 promoter, CloDF13 ori, SmR	Novagen
pKD4	FRT flanked resistance cassette involved vector, oriRγ, KmR	Datsenko and Wanner [42]
pRedET	Red recombinase expression plasmid, repA, pSC101ori, PBAD, gam, beta alpha, recA, tetR	Gene Bridge
707FLP	Flippase expression plasmid, pSC101ori, repA, cl578, FLPe, tetR	Gene Bridge
pZAM	pZA31MCS containing gene *ftfL* from *M. extorquens* AM1	This study
pZAM01	pZAM containing genes *mtdA* and *fch* from *M. extorquens* AM1	This study
pZAM02	pZAM01 containing gene *glyA* from *M. extorquens* AM1	This study
pCDM02	pCDFDuet-1 containing genes *sga*, *hpr* and *gck* from *M. extorquens* AM1	This study
pZSetOH	pZS21MCS containing genes *pdc* from *Z. mobilis* and *adhA* from *L. lactis*	This study
pZSfimCD	pZS21MCS containing genes *fimC* and *fimD* from *E. coli*	This study
pZSydeH	pZS21MCS containing gene *ydeH* from *E. coli*	This study
pZShtrE	pZS21MCS containing gene *htrE* from *E. coli*	This study
pZScsgD	pZS21MCS containing gene *csgD* from *E. coli*	This study

For the initial experiments, bacteria were inoculated at an initial OD (iOD) of 0.7 to solve the low growth issue in the formate M9 minimal medium. However, as EMK02A2 and EMK02A5 showed significantly higher growth rate and formate utilization ability, these strains were inoculated at an iOD of 0.1 and their growth and formate uptake rates were compared with wild-type and EMK02 strains (Fig. 4c, d). The evolved strain EMK02A5 showed strikingly different growth and formate uptake rates under these conditions. To ascertain the ability of EMK02A5 to utilize formate for biomass production, the strain was cultivated with ^13^C-labeled formate. An initial ^13^C-labeling experiment was performed with an iOD of 0.7 and an 8-h cultivation, and the results were compared with those obtained with the EMK02 and EMK02A2 strains (Additional file 1: Figure S1). The strains that underwent longer ALE had a higher proportion of labeled amino acids, which indicated improved formate utilization and biomass production. Then, the fraction of labeled amino acids in the EMK02A5 was measured with an iOD of 0.1, after an 18-h cultivation. The labeled fractions were significantly higher when the iOD was 0.1 (Fig. 4e). This was likely because of lower biomasses at inoculation under these conditions and, therefore, to a lower contribution of unlabeled amino acids from preexisting bacteria. The ^13^C-labeling results clearly showed significantly improved formate assimilation after 150 ALE subcultures.

To verify the production of useful compounds from formate as the main carbon source, the ethanol pathway was overexpressed in the strains. Two genes, *pdc* from *Zymomonas mobilis*, encoding pyruvate decarboxylase (NCBI-Protein ID: AEH63551) and *adhA* from *Lactococcus lactis*, encoding alcohol dehydrogenase (NCBI-Protein ID: NP_267964) were overexpressed [31]. Although ethanol was not detected in the cultures of the EMK02etOH and EMK02A2etOH strains, it was produced by the EMK02A5etOH strain at a concentration of 90 mg/L, after 24-h incubation (Fig. 4f). In addition, a higher portion of labeled ethanol was detected in the fed-batch culture with ^13^C-labeled formate (Additional file 1: Figure S2). This finding confirmed that other useful biochemicals or biofuels can be produced using formate in the absence of sugars.

### Genome sequence analysis of ALE strains

**Fig. 5:**
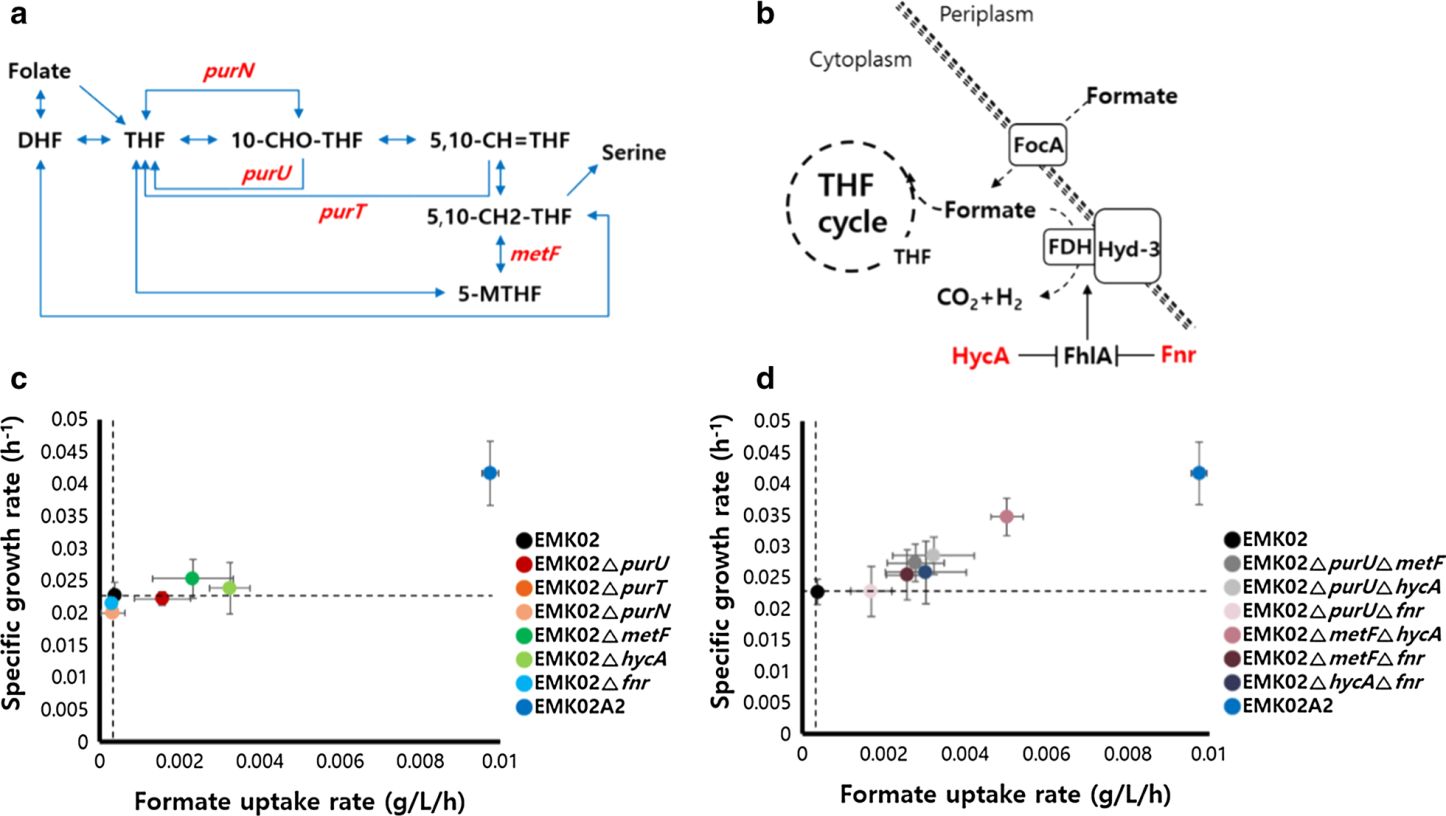
Functional confirmation of the mutations in EMK02A2. The tetrahydrofolate (THF) cycle pathway in *E. coli* (**a**). *purN*, encoding phosphoribosylglycinamide formyltransferase 1; *purU*, encoding formyltetrahydrofolate hydrolase; *purT*, encoding phosphoribosylglycinamide formyltransferase 2; *metF*, encoding 5,10-methylenetetrahydrofolate reductase; DHF, dihydrofolate; THF, tetrahydrofolate; 5-MTHF, 5-methyl-THF. The formate hydrogen lyase (FHL) system (**b**). FhlA is an activator of the FHL system, and FhlA is repressed by HycA and Fnr. Red letters: the deleted genes or proteins. Effects on growth and formate uptake rates by the deletions of one of the THF cycle genes or of the FHL system in EMK02 grown in EMK medium at iOD of 0.1 (**c**). Effects by deletions of two genes of the THF cycle and/or the THF system (**d**)

To gain insights into the phenotype changes in ALE-mutant strains, whole genome DNA sequencing was carried out for the EMK02A2 and EMK02A5 strains. Genome sequencing was conducted twice and only the mutations showing the same results in both sequencing sessions were selected. In addition, only mutations with sequencing quality score above the reference level were considered. When the genome of EMK02A2 was compared to that of the wild-type, *E. coli* BL21 (DE3), 54 mutations were detected. Since *gcvP* had been deleted from the EMK02 genome, this gene was not included in the mutation table. No mutations were detected in the pZAM02 and pCDM02 plasmids. Among the identified mutations, 40 were found to occur in coding regions, including 19 non-synonymous, 19 synonymous, and 2 frameshift mutations (Additional file 1: Table S3). Notably, 90% of these mutations involved seven different metabolic pathways and two individual genes. They are folate metabolism, formate hydrogen lyase regulation, ABC transport, DNA packing, pantothenate and CoA biosynthesis, DNA mismatch repair, stress response, lactate dehydrogenase, and carbamoyltransferase. Each pathway and metabolism are referred to KEGG functional orthologs (KO) and the pathway in KEGG (http://www.genome.jp/kegg/). As folate metabolism is directly associated with the THF cycle of the formate assimilation pathway [15] and formate hydrogen lyase enhances formate consumption [32], we hypothesized that the mutations in the above-mentioned pathways accounted for most of the phenotypic changes observed in the EMK02A2 strain. Among the folate metabolism mutations, a frameshift mutation was found at the first codon of *metF* (Gene ID: 948432 [Genbank]), which codes for methylenetetrahydrofolate reductase and is the rate-limiting enzyme in the THF cycle (Fig. 5a) [27, 33] resulting in a stop codon in the third position. In addition, point mutations were detected in the coding regions of *purU* (Gene ID: 945827 [Genbank]), the formyltetrahydrofolate deformylase; *purT*, (Gene ID: 946368 [Genbank]) and *purN* (Gene ID: 946973 [Genbank]), the phosphoribosylglycinamide formyltransferases (Additional file 1: Table S3). They have major roles in balancing the pools of tetrahydrofolate and 10-formyl tetrahydrofolate for the production of purines [25]. As previously mentioned, *metF* presented a frameshift mutation in EMK02A2, causing loss of function, and non-synonymous mutations were found in *purU*, *purT*, and *purN.* For these reasons, we hypothesized that the mutations in THF cycle-related genes led to improved formate assimilation via an increased availability of 5,10-methylene tetrahydrofolate. In addition, mutations were found in the coding regions of *hycA* (Gene ID: 947193 [Genbank]) and *fnr* (Gene ID: 945908 [Genbank]), which are involved in the regulation of formate hydrogen lyase (Fig. 5b). The latter enzyme converts formate to carbon dioxide and hydrogen, which might be important for the efficient utilization of formate in terms of hydrogen generation and reducing the formate-induced toxic effect. This can be performed by the formate hydrogen lyase complex that consists of two membrane-bound enzymes—formate dehydrogenase-H (FDH-H) and hydrogenase 3 (Hyd-3) [34]. Therefore, we suggested a possibility that the mutations in *hycA* and *fnr* reduced the activities of these genes and increased the expression of formate hydrogen lyase.

To find out if the above mutations actually increased the ability to utilize formate, each of the relevant genes was deleted in the parental strain EMK02. Experiments conducted with a starting iOD of 0.1 in EMK medium demonstrated remarkable improvements of formate utilization and growth rate in the strains EMK02 ∆*purU*, EMK02 ∆*metF*, and EMK02 ∆*hycA* (Fig. 5c). Next, we examined the effects of combined deletions of two genes among *purU*, *metF*, *hycA*, and *fnr*. Among the six mutants, the EMK02 Δ*metF* Δ*hycA* strain grew well in EMK medium and displayed a growth rate as high as half that of the EMK02A2 strain. No significant changes were detected upon additional deletion of *purU* or *fnr* in the EMK02 Δ*metF* Δ*hycA* strain (data not shown).

**Fig. 6:**
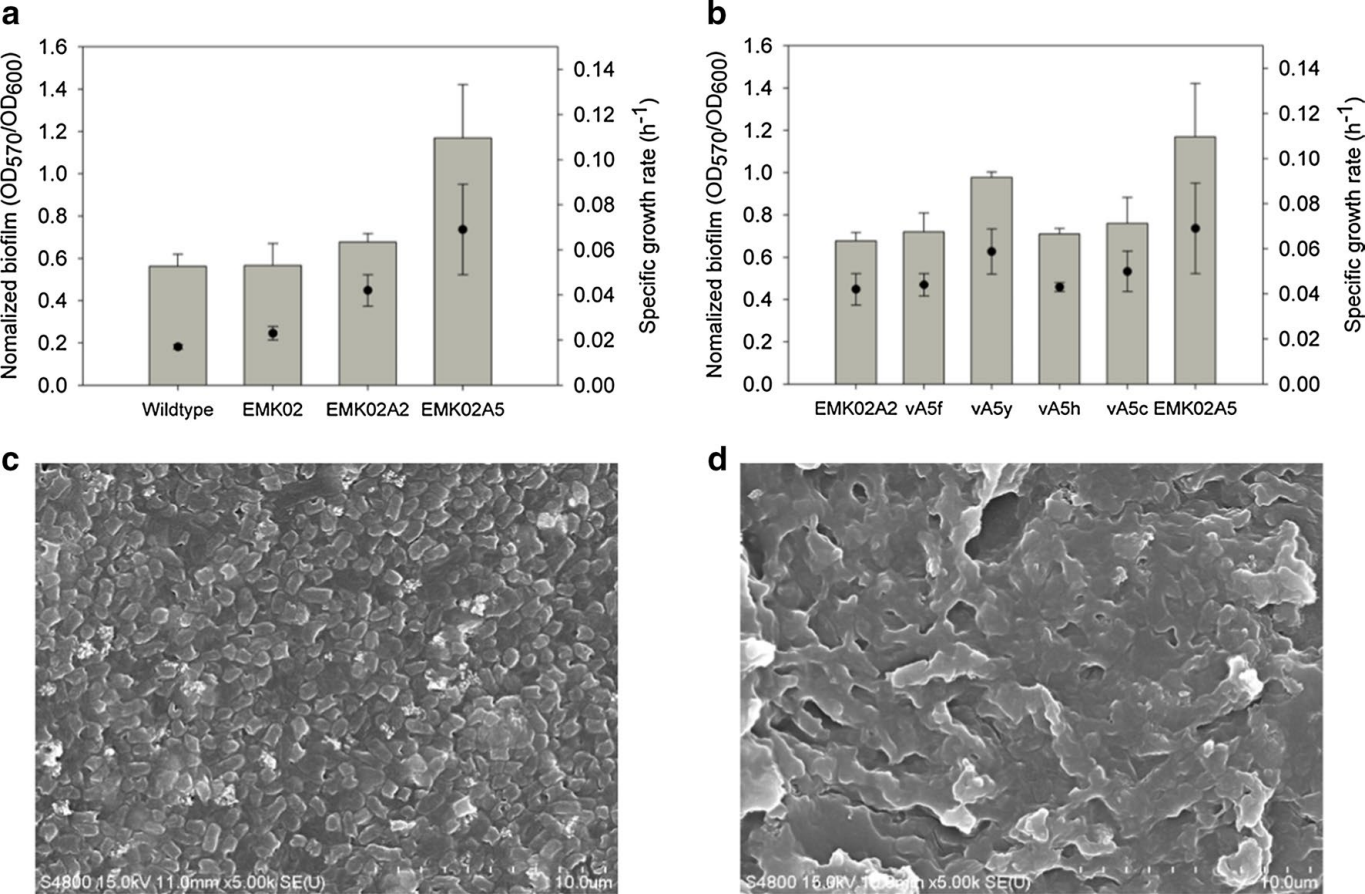
Functional confirmation of mutations in strain EMK02A5. Normalized biofilm formation measured by Crystal Violet assay was compared to the specific growth rates of the strains in EMK medium at iOD of 0.1 among different stages of ALE strains (**a**) and among the strains overexpressing genes related to biofilm formation in EMK02 (**b**). The names, vA5f, vA5y, vA5h, and vA5c, in the *x*-axis represent the strains with overexpression of *fimCD*, *ydeH*, *htrE*, and *csgD*, respectively, in EMK02A2. SEM images showing the biofilm formation of EMK02A2 (**c**) and EMK02A5 (**d**)

A total of 34 mutations were additionally detected in strain EMK02A5 compared to the genome of strain EMK02A2, 23 of which were in coding regions and included 19 non-synonymous mutations, 3 synonymous mutations, and 1 stop-codon gained mutation (Additional file 1: Table S4). The mutations occurring as a result of 90 additional serial subcultures in EMK02A2 were all in the coding regions of the genes involved in seven metabolic pathways. These pathways are related to peptidoglycan biosynthesis, the general secretion pathway, *S*-formylglutathione hydrolase, aldehyde dehydrogenase, diguanylate cyclase, fimbriae metabolism, and flagellar biosynthesis. Among them, diguanylate cyclase, fimbriae metabolism, and flagellar biosynthesis are known to be associated with bacterial mobility and biofilm formation [35]. Biofilm biosynthesis is affected by the metabolism of peptidoglycans (PG), the main component of the cell wall and the biofilm [36], and by the expression of fimbrial proteins, leading to aggregation of bacterial cells [37]. In EMK02A5, non-synonymous point mutations were found in the coding regions of *fimC* (Gene ID: 948843 [Genbank])*, fimD* (Gene ID: 948844 [Genbank])*, htrE* (Gene ID: 944819 [Genbank]), and *flgL* (Gene ID: 945646 [Genbank]). In addition, to form a matrix of bacterial microcolonies, the motility factors must be inhibited. The genes encoding regulators of biofilm formation, *csgD* (Gene ID: 949119 [Genbank]) and *ydeH* (Gene ID: 946075 [Genbank]), also presented non-synonymous point mutations in their coding regions. According to the results from the crystal violet staining assay (CVA), biofilm formation of EMK02A5 was increased by more than twofold compared to that of the EMK02 strain (Fig. 6). This result was in accordance with the results of SEM imaging, which showed increased biofilm formation by the EMK02A5 strain (Fig. 6c, d). It has been reported that biofilm formation is beneficial for bacteria as it may improve their tolerance toward toxic compounds [38–41]. We reasoned that the mutations in the genes related to biofilm formation, i.e., *fimC, ydeH, htrE*, and *csgD*, could account for this effect. Therefore, we individually overexpressed these genes in EMK02A2. All of the resulting strains showed a higher degree of biofilm formation and formate utilization capability, when compared with the original strain (Fig. 6b). Among them, the vA5y strain, overexpressing the *ydeH* gene, proved to be the highest biofilm producer and displayed the strongest ability to utilize formate (Fig. 6b). The results showed that bacterial biofilm formation was closely related to formate utilization and suggested that increased biofilm formation ability was the most important determinant of the phenotypic differences between EMK02A2 and EMK02A5.

## Conclusions

An *E. coli* strain capable of utilizing formate for biomass formation was constructed by overexpression of genes involved in the THF cycle and in serine utilization pathways. Adaptive evolution significantly improved bacterial ability to utilize formate as proven by ^13^C-formate tracing experiments and ethanol production. Genome sequences of the evolved strains allowed us to identify important machineries and pathways related to formate utilization ability, such as the THF cycle, the formate dehydrogenase complex, and biofilm formation. The optimization of these biochemical routes, combined with appropriate strategies of pathway engineering, is expected to generate synthetic *E. coli* formatotrophs.

## Methods

### Strains and plasmids

All strains and plasmids used in this study are presented in Table 1. The bacterial strain *E. coli* BL21 (DE3) was used as a host for constructing the synthetic formatotroph, and *E. coli* DH5α was used for plasmid cloning. The two strains were purchased from KCTC (Daejeon, South Korea) and RBC (Banqiao, Taiwan), respectively.

The genes encoding formate-tetrahydrofolate ligase (*ftfL,* Gene ID: 240007055 [Genbank]), methylene-tetrahydrofolate dehydrogenase (*mtdA,* Gene ID: 240008346 [Genbank]), methenyl tetrahydrofolate cyclohydrolase (*fch*, Gene ID: 240008347 [Genbank]), and serine hydroxymethyltransferase (*glyA*, Gene ID: 240009895 [Genbank]) from *M. extorquens* AM1 were cloned into the pZA31MCS vector (Expressys, Ruelzheim, Germany), whereas the genes encoding serine-glyoxylate transaminase (*sga,* Gene ID: 240008344 [Genbank]), hydroxypyruvate reductase (*hpr*, Gene ID: 240008345 [Genbank]), and glycerate kinase (*gck*, Gene ID: 240009470 [Genbank]) were cloned into the pCDFDuet-1 vector (Novagen, Madison, WI). Other genes related to ethanol production or biofilm formation were cloned into the pZS21MCS vector (Expressys, Ruelzheim, Germany). The genes were amplified using the primers indicated in detail (Additional file 1: Table S1) and the NEB Q5 DNA polymerase and ligated by the Gibson Assembly Master Mix (New England Biolabs, MA, USA). The gene knockout experiment was carried out as previously reported [42], with λ-red recombination using the pRedET transformed strains. PCR products with antibiotic resistance genes were generated by PCR with the primers (Additional file 1: Table S2) and pKD4 as a template, and FLP expression using the 707FLP plasmid (Gene Bridges, Heidelberg, Germany) was used for eliminating the antibiotic resistance genes. All the knockout mutations were confirmed by sequencing of the genomic regions.

### Media and culture conditions

The engineered strains were constructed using Luria–Bertani (LB) medium (10 g/L tryptone, 5 g/L yeast extract, and 10 g/L NaCl), while the mutant strains were cultured in formate M9 minimal medium or EMK medium. The composition of formate M9 minimal medium was 10 mM sodium formate, 0.241 g/L MgSO_4_, 0.011 g/L CaCl_2_, 6 g/L Na_2_HPO_4_, 3 g/L KH_2_PO_4_, 0.5 g/L NaCl, 1 g/L NH_4_Cl, 0.1%(v/v) of 1000× trace elements (27 g/L FeCl_3_·6H_2_O, 2 g/L ZnCl_2_·4H_2_O, 2 g/L CaCl_2_·2H_2_O, 2 g/L Na_2_MoO_4_·2H_2_O, 1.9 g/L CuSO_4_·5H_2_O, and 0.5 g/L H_3_BO_3_) supplemented with 50 μg/mL chloramphenicol, 50 μg/mL kanamycin, 100 μg/mL spectinomycin, 50 μg/mL ampicillin, and 10 μg/mL tetracycline, whenever needed. The medium with 1 g/L glycine and 0.2 g/L yeast extract added to the formate M9 minimal medium is defined as EMK medium. All reagents were purchased from Sigma-Aldrich (St. Louis, MO, USA).

The seed culture was incubated in 5 mL of culture media supplied with 3 g/L yeast extract overnight. The seed strains were pelleted by centrifugation at 3500 rpm for 10 min at 4 °C and washed once with M9 minimal medium. Next, they were resuspended with 50 mL of medium and incubated micro-aerobically in 250-mL flasks sealed with silicone stoppers at 37 °C with shaking at 250 rpm; 0.05 mM IPTG was added at the beginning of culture. For the formate utilization culture with high initial optical density (iOD), 1 g/L glycine was added to the formate M9 minimal medium and the iOD was adjusted to 0.7. For other cultivations, EMK medium was utilized and the iOD was adjusted to 0.1. For fed-batch fermentation, the experiment was conducted with a 3-L fermenter (BioCNS, Daejeon, South Korea) containing 1 L working volume. The cultures were carried out at 37 °C with 150 rpm agitation and 1 vvm air was supplied in the EMK medium.

### Adaptive laboratory evolution (ALE)

For ALE, a total of 150 serial subcultures were carried out once every 24 h in modified EMK medium. The culture medium was diluted after reaching stationary phase. Initially, 1 g/L yeast extract and 5 mM sodium formate were supplied. Every 10 serial subcultures, the amount of yeast extract was gradually decreased, whereas formate was augmented in the culture medium. Then, starting from the 100th subculture, the concentration of formate was fixed at 20 mM and that of yeast extract at 0.2 g/L. Every 30th serial subculture, strain selection was carried out on agar medium containing a high concentration of formate (100 mM formate, 25 g/L LB broth, and 15 g/L agar powder) and the strains that formed large-sized colonies, reflecting efficient formate utilization, were selected.

### Carbon-labeling experiment

For carbon-labeling experiments, 10 mM ^13^C-sodium formate (99% purity; Cambridge Isotope Laboratories, Inc., Cambridge, MA, USA) was added to the medium. Strains were cultured at 37 °C for the times specified in the Results. To extract proteinogenic amino acids, 2–3 mL of culture broth was centrifuged at 13,500 rpm for 10 min at 4 °C. After decanting the supernatant, the cell pellet was frozen using liquid nitrogen and then dried overnight in a freeze dryer (OPERON, South Korea). For the hydrolysis of proteins, the pellets were resuspended in 200 μL of 6 N HCl and placed at 110 °C for 24 h. Then, 200 μL of 6 N NaOH were added and mixed thoroughly. Samples were stored at − 70 °C until they were analyzed by GC–EI-MS. Sample preparation and GC–EI-MS analysis were carried out as previously reported [22]. The metabolite samples underwent chemical derivatization with *N*-methyl-*N*-*tert*-butyldimethylsilyltrifluoroacetamide (Sigma-Aldrich, St. Louis, MO, USA) for GC-EI-MS analysis, and were analyzed using a Bruker 450-GC instrument coupled with a Bruker 300-MS single quadrupole mass spectrometer (Bruker Inc. Fremont, CA, USA).

### Whole genome sequencing

Genomic DNA was purified from wild-type and ALE-mutant strains using a Wizard Genomic DNA Purification Kit (Promega, Madison, WI, USA). The DNA library was prepared using a TruSeq DNA PCR-free kit (Illumina, Inc., San Diego, CA, USA). The sequencing of gDNA was carried out by Macrogen (Daejeon, South Korea) using an Illumina Hiseq4000 platform (Illumina, San Diego, CA, USA). The overall sequencing was performed according to Macrogen’s standard protocols (https://dna.macrogen.com).

### Analytical methods

The optical density was measured using a UV–VIS spectrophotometer (model DU-730; Beckman Coulter Inc., Fullerton, CA, USA). The metabolite analysis in the supernatants was carried out by high-performance liquid chromatography (HPLC) using a Waters 2414 refractive index detector (Waters Corp, Waltham, MA, USA) equipped with a Shodex SH1011 column (Shodex, Tokyo, Japan). The column temperature was 75 °C, and 10 mM sulfuric acid was used for the mobile phase at a flow rate of 0.6 mL/min. The biofilm was detected using a Crystal Violet assay (Sigma-Aldrich, St. Louis, MO, USA) following the manufacturer’s protocol [23] and also analyzed by scanning electron microscopy (SEM, Hitachi S-4700, Tokyo, Japan).

## Supplementary information

**Additional file 1. Table S1.** Oligonucleotides used for gene cloning in this study; **Table S2.** Oligonucleotides used for gene deletion in this study; **Table S3.** Mutations in the EMK02A2 strain compared to EMK02; **Table S4.** Mutations in the EMK02A5 strain compared to EMK02A2; **Figure S1.** Carbon labeled experiment with ALE mutants; **Figure S2.** The proportion of labeled ethanol in the EMK02A5 strain after 24-h incubation.

## Data Availability

The datasets used and/or analyzed during the current study are available from the corresponding author on reasonable requests.

## Abbreviations

ALE: adaptive laboratory evolution
iOD: initial optical density
THF: tetrahydrofolate
N^10^-fTHF: 10-formyl tetrahydrofolate
5,10-CH^+^-THF: 5,10-methenyl tetrahydrofolate
5,10-CH_2_-THF: 5,10-methylene tetrahydrofolate
FtfL: formate-tetrahydrofolate ligase
Fch: methenyl tetrahydrofolate cyclohydrolase
MtdA: methylene-tetrahydrofolate dehydrogenase
GlyA: serine hydroxymethyltransferase
PGA: phosphoglyceric acid
Sga: serine-glyoxylate transaminase
Hpr: hydroxypyruvate reductase
Gck: glycerate kinase
2PGA: 2-phosphoglycerate
PEP: phosphoenolpyruvate
OAA: oxaloacetate
CVA: crystal violet staining assay
FDH-H: formate dehydrogenase-H
Hyd-3: hydrogenase 3
