# Supplementary material for: Adaptively evolved Escherichia coli for improved ability of formate utilization as a carbon source in sugar-free conditions
Source: Biotechnol Biofuels. 2019 Sep 3;12:207. doi: 10.1186/s13068-019-1547-z (PMC6720381; doi:10.1186/s13068-019-1547-z)
Supplement: Supplementary file 1 — Additional file 1. Table S1. Oligonucleotides used for gene cloning in this study; Table S2. Oligonucleotides used for gene deletion in this study; Table S3. Mutations in the EMK02A2 strain compared to EMK02; Table S4. Mutations in the EMK02A5 strain compared to EMK02A2; Figure S1. Carbon labeled experiment with ALE mutants; Figure S2. The proportion of labeled ethanol in the EMK02A5 strain after 24-h incubation. [file 13068_2019_1547_MOESM1_ESM.docx]

Additional Information

Adaptively evolved *Escherichia coli* for improved ability of formate utilization as a carbon source in sugar-free conditions

Seung-Jin Kim^1^, Jihee Yoon^1^, Dae-Kyun Im^1^, Yong Hwan Kim^2^, Min-Kyu Oh^1,^*

^1^ Department of Chemical and Biological Engineering, Korea University, Seongbuk‑gu, Seoul 02841, Republic of Korea.

^2^School of Energy and Chemical Engineering, UNIST Ulsan, 44919, Republic of Korea

^*^Corresponding author. M.-K. Oh. [mkoh@korea.ac.kr](mailto:mkoh@korea.ac.kr)

First Author. S.J. Kim. [tmdwls7526@naver.com](mailto:tmdwls7526@naver.com)

Co-author. J.Yoon. [yoonz19@daum.net](mailto:yoonz19@daum.net)

Co-author. D.K. Im. [meganti@korea.ac.kr](mailto:meganti@korea.ac.kr)

Co-author. Y.H. Kim. [metalkim@unist.ac.kr](mailto:metalkim@unist.ac.kr)

**Table S1.** Oligonucleotides used for gene cloning in this study

| Primers | Sequence |
| --- | --- |
| *ftfL*_fw | 5’- attaaagaggagaaaggtacTGAGAGAAATGCCCTCAG |
| *ftfL*_rv | 5’- acatgattcccctcctGAGAGCAGCGGCTAGAAC |
| *mtdA* and *fch*_fw | 5’- cgctgctctcaggaggGGAATCATGTCCAAGAAGC |
| *mtdA* and *fch*_rv | 5’- gctcatcctttcctcctCGATCGGCTCAGTTTACC |
| *glyA*_fw | 5’- gagccgatcgaggaggaaaggATGAGCGCCGGAACTGCG |
| *glyA*_rv | 5’- ctcgagggggggcccggtacTCAGGCGTAGATCGGGAACC |
| *sga*_fw | 5’- tcgagctcggcgcgcctgcaaggaggAAATTCTCATGGCGGCAAC |
| *sga*_rv | 5’- cgcaagcttgtcgacctgcaAAAGAATGAGGCCCCAAG |
| *hpr*_fw | 5’- actttaataaggagatatacGGGGTCCAGAATGACAAAG |
| *hpr*_rv | 5’- ccttgatgcgcctcctGTTTCTTACGCCTCGACG |
| *gck*_fw | 5’- cgtaagaaacaggaggCGCATCAAGGTGGTCGAG |
| *gck*_rv | 5’- tgatggtgatggctgctgccCTGCATGAAGGGTCAGAC |
| *pdc*_fw | 5’-atgtcaaacaaaccctttatctaccaggcacctttcccgatggggaaagacaataccgaagtgtaggctggagctgcttcg |
| *pdc*_rv | 5’-ttacttagtgcagttcgcgcactgtttgttgacgatttgctggaagaagtcgttacctttattccggggatccgtcgacc |
| *adhA*_fw | 5’-tataaatgcactttgcgtgccg |
| *adhA*_rv | 5’-gcaagcgtgaaattacaatcgc |
| *fimCD*_fw | 5’- attaaagaggagaaaggtacTGATAACAGGAACAGGACAG |
| *fimCD*_rv | 5’- gctgcaggaattcgatatcaCTCATCACGCCCCCTTAAC |
| *ydeH*_fw | 5’- attaaagaggagaaaggtacaaaATGATCAAGAAGACAACG |
| *ydeH*_rv | 5’- ctcgagggggggcccggtacTCAATGAATGTTAAACGGAG |
| *htrE*_fw | 5’-attaaagaggagaaaggtacGCAGGAAGCATAGCGTGAC |
| *htrE*_rv | 5’-ctcgagggggggcccggtacTTACTGAATCTGACACCGAATTC |
| *csgD*_fw | 5’-attaaagaggagaaaggtacAAAAAGCGGGGTTTCATC |
| *csgD*_rv | 5’-ctcgagggggggcccggtacGGCTTTATCGCCTGAGGTTATC |

**Table S2.** Oligonucleotides used for gene deletion in this study

| Primers | Sequence |
| --- | --- |
| △*gcvP*_fw | 5’-  aacatatccgccgtgagaaagcgaactccaacatttgtacttcccaggta gtgtaggctg gagctgcttc |
| △*gcvP*_rv | 5’-CTGTTCCGCTTTCGCGCGCAGATCAGTCAGATCGATGTTGCCGTTTTTATGTCCATATGAATATCCTCCT |
| △*purU*_fw | 5’- ATGCATTCACTCCAACGTAAAGTTCTGCGTACTATTTGTCCGGACCAAAAGTGTAGGCTG GAGCTGCTTC |
| △*purU*_rv | 5’- TAATCGTCCGATTACCGTAAACAAAGACGCGCTGTGCCAGTACTTTGTAT GTCCATATGAATATCCTCCT |
| △*purT*_fw | 5’- TGGACGCGTGAAGACCCTGCATCCGAAAGTACATGGTGGCATTCTG  GGCCGTGTAGGCTGGAGCTGCTTC |
| △*purT*_rv | 5’- ACCCTGAACCTGGGTTGCGGTAGCAACGGAGGCTTCTTTCACATTCTCTTGTCCATATGAATATCCTCCT |
| △*purN*_fw | 5’- AAATTAAAGGCACCGTACGGGCAGTTTTCAGCAATAAGGCCGACGCGTTCGTGTAGGCTGGAGCTGCTTC |
| △*purN*_rv | 5’- CCAGAAAAGGGGGAAAAAAACTGCTGGAGCTTGCGATTTACAGGTGAATTGTCCATATGAATATCCTCCT |
| △*metF*_fw | 5’- TGCACAACACAACATATAACTACAAGCGATTGATGAGGTAAGGTATGAGCGTGTAGGCTGGAGCTGCTTC |
| △*metF*_rv | 5’- TTCACAAAAGCCACACTATTTATAAACCAGGTCGAACCCCCAGCGTATGGGTCCATATGAATATCCTCCT |
| Δ*hycA_*fw | 5’- CATCGCACAGCGGCATCGTCGCCTACAGGACCAGTGGCACATCTACTGCA GTGTAGGCTG GAGCTGCTTC |
| Δ*hycA­*_rv | 5’- GCCTCAGCTCATGCTGCCGGGCTTTGTCCCTTTACCAGTTGGCTTAAATT GTCCATATGAATATCCTCCT |
| Δ*fnr*_fw | 5’- gaaaagcgaattatacggcgcattcagtctggcggttgtgctatccattg gtgtaggctg gagctgcttc |
| Δ*fnr*-rv | 5’- agcgcatcgttattttcgatggtgatgtatttacctttgactgccagcat gtccatatgaatatcctcct |

**Table S3.** Mutations in the EMK02A2 strain compared to EMK02

| Position | Reference | Alternate | Mutation Type | Genes | Residue change |
| --- | --- | --- | --- | --- | --- |
| 87220 | G | A | synonymous | *leuO* | K16K |
| 145903 | T | C | synonymous | *yadG* | G107G |
| 152354 | G | GT | frameshift | *panB* | R18fs* |
| 272929 | T | C | intergenic |  |  |
| 352886 | A | G | non-synonymous | *tauC* | H12R |
| 355632 | T | C | intergenic |  |  |
| 573197 | T | C | intergenic |  |  |
| 592514 | G | C | intergenic |  |  |
| 601413 | A | G | synonymous | *uspG* | V93V |
| 689755 | A | T | synonymous | *rhsC* | I102I |
| 768867 | G | T | non-synonymous | *nu1* | G70V |
| 812571 | A | G | non-synonymous | *ybhF* | R133G |
| 850458 | G | A | intergenic |  |  |
| 1211439 | C | T | non-synonymous | *ymgJ* | A8V |
| 1247163 | T | C | synonymous | *ychH* | R77R |
| 1275980 | G | C | non-synonymous | *purU* | S144T |
| 1384023 | T | C | synonymous | *fnr* | G222G |
| 1384350 | T | G | synonymous | *fnr* | V113V |
| 1417424 | C | T | non-synonymous | *ldhA* | P325S |
| 1440176 | G | T | synonymous | *bhsA* | A23A |
| 1573618 | A | C | non-synonymous | *ydeI* | N57T |
| 1616189 | T | A | intergenic |  |  |
| 1877133 | G | A | non-synonymous | *purT* | E179K |
| 2137719 | T | C | intergenic |  |  |
| 2239728 | T | C | synonymous | *InaA* | C36C |
| 2487189 | A | T | non-synonymous | *purN* | T161S |
| 2528862 | G | A | synonymous | *trmJ* | P87P |
| 2558910 | T | C | synonymous | *purL* | P817P |
| 2677075 | G | C | synonymous | *hypF* | P104P |
| 2690002 | T | C | non-synonymous | *hycA* | I132T |
| 2742929 | T | C | non-synonymous | *mazG* | C114P |
| 2745583 | T | C | synonymous | *relA* | L218L |
| 2746742 | T | G | non-synonymous | *rlmD* | L281W |
| 2808596 | G | A | synonymous | *mutH* | A220A |
| 2988396 | C | T | intergenic |  |  |
| 3400181 | C | A | intergenic |  |  |
| 3463600 | C | T | synonymous | *ftsX* | L288L |
| 3481772 | C | G | non-synonymous | *rhsB* | A68G |
| 3486919 | T | C | non-synonymous | *yhhI* | L55P |
| 3512536 | A | G | non-synonymous | *arsB* | I408V |
| 3512652 | C | T | synonymous | *arsC* | C12C |
| 3514641 | A | G | non-synonymous | *yhiS* | K324R |
| 3515361 | G | T | non-synonymous | *slp* | V74L |
| 3517392 | C | T | synonymous | *hdeB* | L28L |
| 3684918 | G | A | non-synonymous | *dinD* | R125K |
| 3750787 | T | C | synonymous | *glvA* | L24L |
| 3769514 | C | T | synonymous | *recF* | R192R |
| 3797199 | C | T | intergenic |  |  |
| 3903913 | T | C | intergenic |  |  |
| 4022076 | G | C | non-synonymous | *fpr* | D105H |
| 4041158 | T | TT | frameshift | *metF* | M1fs* |
| 4123296 | C | A | intergenic |  |  |
| 4261979 | C | T | intergenic |  |  |
| 4275717 | T | A | intergenic |  |  |

* fs: frameshift

**Table S4.** Mutations in the EMK02A5 strain compared to EMK02A2

| Position | Reference | Alternate | Mutation Type | Genes | Residue change |
| --- | --- | --- | --- | --- | --- |
| 157875 | A | G | non-synonymous | *htrE* | K133E |
| 168830  170169 | T  G | C  GA | synonymous  intergenic | *mrcB* | L814L |
| 347564  355805  689379 | T  C  A | C  A  C | non-synonymous  non-synonymous  intergenic | *frmB*  *ykiB* | L46L  P64Q |
| 740253 | TC | C | intergenic |  |  |
| 747017 | G | A | intergenic |  |  |
| 766431  788230 | A  G | C  C | non-synonymous  non-synonymous | *borD*  *orf_401* | K97Q  V38L |
| 891018 | C | T | synonymous | *tfaE* | V20V |
| 1097421 | A | T | non-synonymous | *ycdT* | K90I |
| 1105297 | T | C | non-synonymous | *CsgD* | V154A |
| 1143039  1170003  1478490 | C  G  C | A  T  T | non-synonymous  intergenic  intergenic | *flgL* | A149D |
| 1545542 | C | A | non-synonymous | *fimD* | P393T |
| 1572602 | C | T | non-synonymous | *ydeH* | T180I |
| 1582513 | A | C | non-synonymous | *ydfK* | K62T |
| 1823035 | T | C | non-synonymous | *yeaP* | V317A |
| 1910305  2017589 | G  C | A  A | non-synonymous  intergenic | *flhA* | V220I |
| 2198184 | T | C | intergenic |  |  |
| 2802210  2998771 | T  C | C  T | non-synonymous  stop gained | *ppdA*  *yghQ* | V81A  E356sg |
| 3105615 | A | T | synonymous | *alx* | G320G |
| 3325817  3482641  3482984 | G  C  A | A  T  G | non-synonymous  non-synonymous  non-synonymous | *gspO*  *rhsB*  *rhsB* | G16D  R358C  N472S |
| 3622828  3934181 | A  A | G  C | non-synonymous  intergenic | *aldB* | T23A |
| 4233361  4451962 | G  A | T  C | Intergenic  intergenic |  |  |
| 4453737 | T | A | non-synonymous | *fimC* | V54E |

**
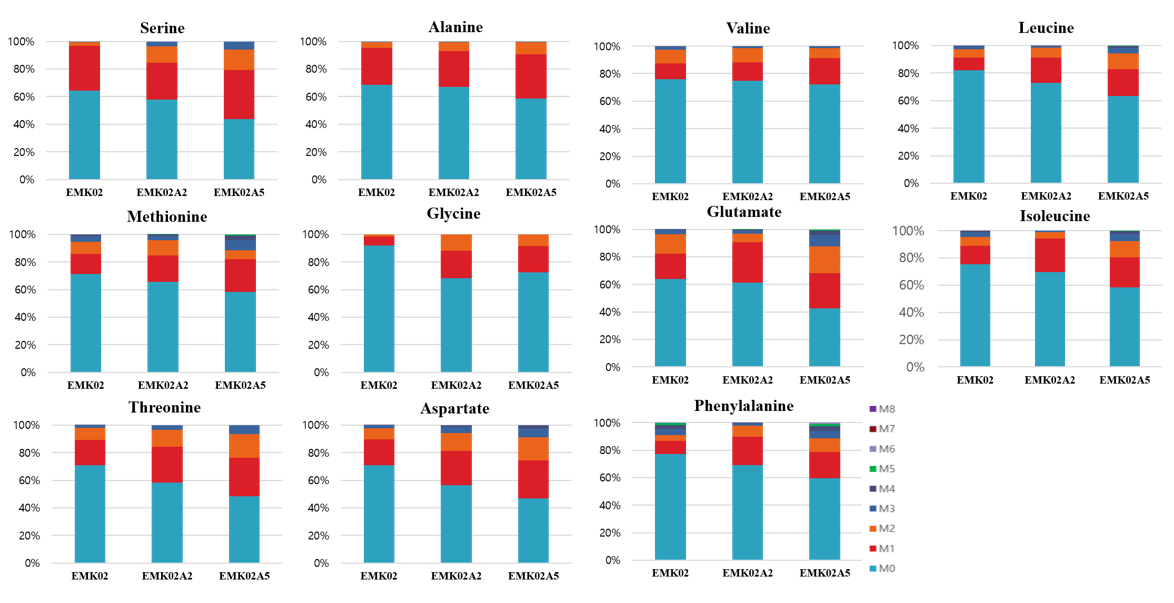
**

**Figure S1**. The proportion of labelled amino acids in evolved mutant strains at 8 hours after the addition of ^13^C-labelled formate in formate minimal medium supplied with 1 g/L glycine with initial OD of 0.**7**. M0-M8 denotes incorporating number of carbon isotope atom in proteinogenic amino acids.


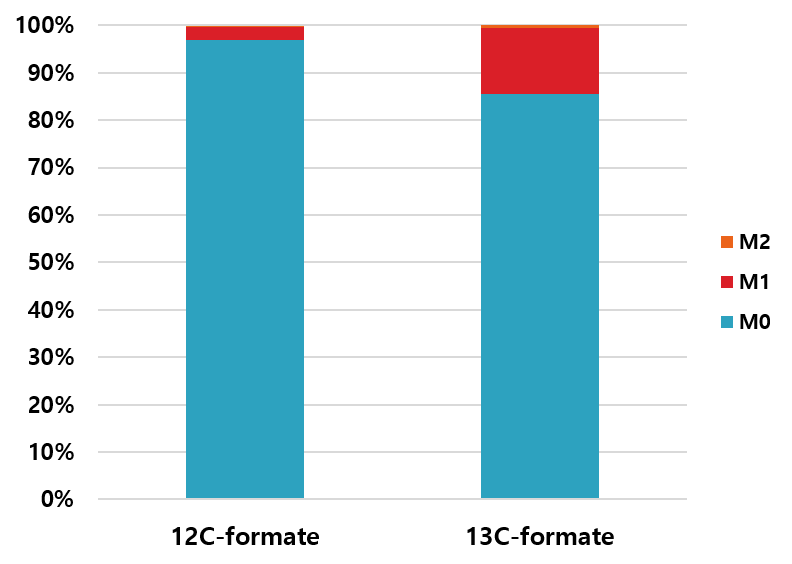


Figure S2. The proportion of labelled ethanol in the EMK02A5 strain after 24-h incubation with unlabeled formate or ^13^C-labelled formate in M9 minimal medium supplied with 1g/L glycine and 0.2g/L yeast extract at iOD of 0.1. M0-M2 denotes incorporating number of carbon isotope atom in ethanol.
